# The Role of Communication Affordances in Post-Traumatic Stress Disorder Facebook and WhatsApp Support Groups

**DOI:** 10.3390/ijerph18094576

**Published:** 2021-04-26

**Authors:** Daphna Yeshua-Katz

**Affiliations:** Department of Communication Studies, Ben-Gurion University of the Negev, Beer-Sheva 8410501, Israel; yeshuad@bgu.ac.il

**Keywords:** stigma, social media, affordances, social support, post-traumatic stress disorder

## Abstract

(1) Background: Digital health research has indicated that people with stigmatized health problems are drawn to online support groups (OSGs) because these groups help them to manage such conditions. However, little is known about how media affordances—interactions between the technology and the user—reconfigure the ways in which stigmatized individuals use OSGs and interact with others like themselves. (2) Method: The current study applied an affordance framework to evaluate how Facebook and WhatsApp support groups can help military veterans and their partners cope with post-traumatic stress disorder (PTSD) and was based on interviews with 34 PTSD-OSG members in Israel. (3) Findings: This research identified five affordances that members appraised as enhancing their coping efforts in the digital world: visibility, availability, multimediality, surveillance, and synchronicity. (4) Conclusions: This study reveals the connection between a specific stigmatized mental health disorder (i.e., PTSD) and perceptions of communication technologies (i.e., affordances), and specifies the uses of technologies for coping with this mental health disorder. Moreover, this study may inform digital intervention designers about which communication affordances can potentially lead to better health outcomes.

## 1. Introduction

Social media applications now serve as the primary online connection for most individuals. In recent years technologies such as Facebook, Twitter, and WhatsApp have become significant social support resources [1]. Online support groups (OSGs) are growing areas of communication research. This trend is illustrated by the growing number of systematic reviews examining OSGs across various health topics [2,3,4]. The role of computer-mediated communication (CMC) appears to be especially important for individuals coping with stigmatized mental health issues. Several studies have highlighted the fact that people with stigmatized health problems are drawn to OSGs because these groups help them to manage their stigmatized health conditions [5,6,7,8,9,10].

However, little is known about how media affordances—the outcomes of the relationship between the object/technology and the user [2,11,12]—reconfigure the ways in which stigmatized individuals use OSGs and interact with others like themselves. We do not know, for example, how the visibility of the person who is in OSGs affects the motivation of stigmatized individuals to openly become members of these groups and contribute content to them.

Therefore, we carried out a case study of a stigmatized health condition OSG—specifically, a post-traumatic stress disorder (PTSD) OSG. In this study, we inquired as to how the OSG members in question—Israeli veterans and veterans’ spouses—perceived Facebook and WhatsApp OSG affordances in terms of how they lined up with their/their partners’ efforts to cope with PTSD-related distress. This study highlights the role of communication technologies in the coping process by focusing on the opportunities and challenges they provide to responding to distress. Examining communication technologies from the perspective of people who cope with PTSD will help healthcare professionals, researchers, and digital intervention designers understand the uses and effects of communication technologies by people with PTSD in particular and with marginalized identities in general.

### 1.1. Online Support for Stigmatized Individuals

Individuals with marginalized identities who do not have much offline social support may turn to OSGs to compensate for the lack of such resources in their physical environments. In the context of various health conditions, people with mental health disorders are more likely than others to use online support groups [13]. Moreover, a nationally representative sample of adults indicated that the more they reported having social stigma concerns, the more likely they were to seek online support instead of an in-person support group or traditional treatment [5].

Online support groups offer people who are coping with stigmatized health issues various benefits. First of all, they can find both informational and emotional support [1,3,14], and they can receive stand-alone or complementary informal support [14,15]. Members of OSGs can also make use of the specific practical opportunities afforded by these groups: that is, OSGs are presumably accessible and affordable, and online forums can provide a safe and anonymous environment in which to hear and be heard (although this is less true of public groups on social media) [16]. Finally, OSGs are beneficial as they are not location-specific [14]. Stigmatized individuals can reap the benefits of joining a group of others who are like them: they can feel less isolated and less different, disclose a secret part of their lives, share experiences and learn from the experiences of others, and receive empathy. Stigmatized individuals make use of various media platforms, but less is known about how media affordances may determine the ways in which these individuals utilize OSGs. 

### 1.2. Communication Affordances

The idea of affordances stems from ecological psychology and is based on how individuals perceive the objects in their environment: both about what the object is and what potential uses it affords [12]. Communication affordances are defined as the outcomes of the relation between the technology and the user [2,11,12]. As Coulson [17] indicated, the emphasis in the theory is on the *interaction* between the user and the object/technology and its resulting outcomes. In this view, affordances are not exclusively the properties of people; they are created in the interplay between technology, human users, and their varied contexts [18]. A distinction can be drawn between affordances and features. Whereas features focus on the technical dimensions of technology, such as taking photos with a smartphone and sharing them, an affordance concerns how such features are interpreted and acted on by users, such as the ability to document aspects of life.

Computer-mediated communication researchers [11,17,19,20,21,22,23] have used the affordance approach to examine how CMC creates new opportunities for, or challenges to, communication in mediated environments. Rains [2] recently introduced a digital coping model that illustrates how people use communication technologies to cope with distress resulting from illness. The model presents the process by which the appraisal of common stressors faced by people who are ill, together with the assessment of technological affordances, leads to engaging in digital coping. Rains [2] presents six types of affordance in his model: availability, control, diversity, documentation, reach, and visibility. In his model, Rains [2] stresses the role of social predictors that might cause communication affordances to be salient among patients. One of these predictors is stigma. Specifically, Rains [2] contends that by making patients wary of potential reprisals for their condition, stigma could make salient the online affordance of anonymity. However, these days, the assumption of anonymity is no longer valid. Online support groups active on blogs and discussions used to offer anonymity and enabled online communication under a pseudonym [24]. However, newer-generation OSGs operating on platforms such as Facebook result in high visibility levels and make personal information public by exposing users’ friends, location, and photos. 

This shift from anonymity in older-generation OSGs to visibility in newer-generation OSGs calls for an application of the affordance approach to the study of stigmatized individuals’ motivations to become members of PTSD-OSGs. Using the affordance approach in this study benefits the building of theoretical knowledge in at least two ways: first, an affordance approach provides a scientific framework for selecting a social media platform. With this approach, we can identify the critical functions that OSGs should have, link them to behavioral or health-related theories, and inform OSG design [25]. 

Second, rather than relying on a single platform that later becomes obsolete, this approach goes beyond the selection of OSGs based on a media platform’s popularity. An affordance approach encourages researchers to look at communication enabled by the relationship between OSG members’ communicative practices and a particular technology’s material functionality. In other words, this approach focuses on the question of what combination of material features enables stigmatized individuals to do the things that had been challenging or impossible for them to do without the technology and vice-versa (i.e., which material features hamper stigmatized individuals’ technology use) [26,27]. Hence, we can use a PTSD-OSG case study to describe the affordances that stigmatized individuals perceive as helpful or harmful and present potential platforms that would meet the relevant criteria.

### 1.3. Coping with PTSD 

Post-traumatic stress disorder is a mental health disorder that some people develop after experiencing or witnessing a life-threatening event, such as combat, a natural disaster, a car accident, or sexual assault [28]. Many soldiers returning from war with PTSD may be reluctant to seek help; individuals with PTSD symptoms may be concerned that they will be considered weak or stigmatized and, thus, resist seeking treatment (for a review, see [29]). Post-traumatic stress disorder is characterized by various symptoms. The most prominent are re-experiencing symptoms (e.g., flashbacks, nightmares); avoidant symptoms, including avoiding thoughts, feelings, or situations that bring back memories of the trauma; and hyperarousal symptoms, such as sleep problems and hypervigilance [28].

Research has confirmed that cohabiting partners and spouses play a central role in veterans’ mental health and rehabilitation [30]. However, post-traumatic stress symptoms are likely to be associated with veterans’ feelings of loneliness, which may negatively affect post-combat marital adjustment [31]. Living with a veteran with PTSD can affect a veteran partner’s psychological well-being and health outcomes; the nature of combat-related PTSD places a significant burden and responsibility on partners. Cohabiting partners struggle with interpersonal relations, emotional turmoil, and barriers in caring for themselves and the individual with PTSD [32,33,34,35].

One way to reduce the impact of these stressors is through engaging in communication-focused coping strategies, including seeking and gaining social support [36,37,38]. A content analysis of internet discussion forums for female partners of male veterans with combat-related PTSD revealed themes discussed in the groups such as the all-consuming effect of the illness, walking on eggshells, ambiguous loss, aloneness, and facing PTSD as a unit. The use of Facebook support groups for military families results in supportive outcomes, including seeking and providing support and relational maintenance and development. Nevertheless, OSG use also results in unsupportive outcomes such as privacy concerns, gossip, and support network breakdown [39]. Given the potential barriers to support that veterans and their partners face when coping with PTSD and the role social media can have as a resource for social support, a PTSD-OSG conducted on social media may be especially important to this population.

### 1.4. Case Study: PTSD Support Groups for Israeli Veterans and Their Partners on Facebook and WhatsApp

This study aimed to examine the way members of PTSD-OSGs—veterans and cohabiting partners—perceived the media affordances of their Facebook and WhatsApp support groups. Israel is a good context in which to conduct an academic inquiry into PTSD-OSGs for two reasons: First, the rates of social media usage in Israel are incredibly high. As of January 2020, 91% of Israel’s adult population used WhatsApp, and 85% used Facebook and belonged to an average of 16 Facebook groups [40], compared with 69% of the American adult population [41]. Second, although Israel contends on an ongoing basis with political violence, a relatively large number of veterans with PTSD and their families do not receive professional help. Levi and Lubin [42] examined highly detailed medical records of the Israel Defense Forces (IDF) and the Israeli Ministry of Defense and documented a gap of anywhere between 3% and 11% between treatment-seeking by IDF veterans following war deployment and the actual prevalence of PTSD in this soldier population.

Moreover, those who receive professional psychiatric help from the defense establishment’s rehabilitation services face grim psychosocial consequences. A report funded by the Ministry’s Disabled Veterans Department [43] revealed that 75% of this group suffers from social isolation (in contrast to 30% of the sample of disabled veterans with no diagnosed mental disorders), 43% (vs. 14%) report that they have no friends, and 14% (4%) do not leave their homes. Only 30% (60%) are employed. Nevertheless, this same report revealed that 92% of this group use the internet (vs. 97%). 

Three aspects make the study of digital affordance roles in Israeli PTSD-OSGs theoretically and empirically critical: (1) the lack of evidence about how stigmatized individuals perceive OSG communication affordances on social media, (2) the extensive use of Facebook and WhatsApp in Israel and the potential of these platforms for forging connections among the socially isolated, and (3) the lack of official support for Israeli veterans with PTSD and the social isolation they experience. To examine the role of Israeli PTSD-OSG affordances, this study posited the following research question:

RQ: How do Israeli army veterans and veterans’ partners perceive the role of digital affordances in their online support groups?

## 2. Methodology

The current study is part of a larger research project that examined the role of PTSD-OSGs for military veterans and their partners. The study employed a mixed-method approach that included qualitative in-depth interviews and survey methods. Ethical approval was obtained from the Ben-Gurion University Human Research Ethics committee (ID: 1640-1). 

This paper presents findings from in-depth interviews with 34 members of PTSD-OSGs (see Table 1 for participant information). These interviews captured participants’ voices as they told their stories, creating detailed representations of their experiences in PTSD-OSGs. This method facilitated an understanding of the motivations to join an online support group and the role that technological affordances played in deciding to join and become active members of these groups. 

Data collection progressed in two stages. First, the author identified and selected PTSD-OSGs for Israeli veterans and their partners and interviewed the groups’ administrators (hereafter, admins). The author selected these OSGs through preliminary background interviews with representatives of PTSD veterans’ associations and confirmed that members had actively contributed messages to them during the previous six months. Then, the admins distributed invitations for an interview in their respective groups on Facebook and WhatsApp. The invitation disclosed that the goal was to study PTSD-OSGs and invited participants to volunteer for the study. The author and two research assistants conducted and audio-recorded in-depth interviews either face-to-face (*n* = 27), by phone (*n* = 5), or on Skype (*n* = 2). Participants were assigned pseudonyms, and recordings of the interviews were erased after transcription to ensure participants’ confidentiality. The semi-structured interviews ranged from 40 to 90 minutes in duration (mean = one hour). The interview protocol addressed themes such as motivations to join the OSGs, experiences in the different PTSD-OSGs, and the role of PTSD-OSG affordances. Interviewees filled out a short demographic questionnaire before the start of the interview.

The study included 34 participants (21 men and 13 women) aged 29–77 (mean = 47) (see Table 1 for participant information). All participants were either members of WhatsApp or Facebook PTSD-OSGs or both. Twenty participants reported that they were coping with PTSD; nine were female partners of men who were coping with PTSD; and one participant was a group facilitator. 

### Analysis

To identify the themes emerging from the interviews, we conducted an inductive thematic analysis [44,45]. The research team initially coded the 34 PTSD-OSG members’ interviews to identify their perception of PTSD-OSG affordances. This process resulted in 30 unique codes related to the core topics of incentives to join online support groups, reasons behind the choice of a digital platform, experiences in the group, and interactions with other group members. The research team reanalyzed the coded data to produce 13 thematic categories. The 13 thematic categories were further reduced to five underlying concepts—visibility, availability, multimediality, surveillance, and synchronicity—all central to the communication affordances framework [11,17,19,20,21,23] (see Table 2 for thematic categories).

## 3. Findings

In their search for online support, participants pointed to five essential technological affordances that played a role in their decision about which medium to use. These affordances were visibility, availability, multimediality, surveillance, and synchronicity. 

### 3.1. Visibility

The potential visibility of online content played a significant role in deciding which digital platform to use. Although most participants already used multiple groups on Facebook and WhatsApp, most found WhatsApp to be a safer space for support. They perceived the WhatsApp group as a closed space that guaranteed a low level of visibility and less risk of online disclosure. Participants felt that this low visibility safeguarded their online content from being posted outside the boundaries of the group. In the words of Chaim: “[Group admin name] once said about the WhatsApp group, ‘What happens in the group, stays in the group. Don’t share what’s being said there.’” And another participant, Ben, said:

“Facebook is problematic. My profile is connected to my business, and it’s in a public place. Google searches will bring up your Facebook posts, even from closed groups, sentences, you won’t be able to log in to see the entire post, but you’ll see the subject heading. I am cautious with Facebook. WhatsApp—a little easier.”

The high visibility of Facebook posts caused some groups to be inactive. Gershon, an admin of an active WhatsApp group, said: “In addition to our WhatsApp group, we also started a Facebook group, but there was no activity there. People don’t use this platform. Maybe because of the fear that other people can see it too.” Nava, an avid user of the women’s PTSD support group, preferred not to use Facebook out of fear that someone would repost her posts: “If I post a picture on the Facebook group, let’s say with my husband, why do I need the whole world to know? *Here is her husband who has PTSD*”.

The low level of visibility and perceived anonymity of WhatsApp motivated some members to disclose, as Rachel explained: “Women are afraid to write very openly on Facebook. Someone might take pictures, copy them. On WhatsApp, women get right down to business. They share personal stuff immediately, and as soon as they join the group, they ask for advice”. As per the participants’ above descriptions, most perceived WhatsApp’s lower visibility level as affording a safe space to share their most vulnerable moments with group members. Although most of them valued this low visibility, five found it problematic. Some felt it prevented them from getting to know the other group members, and others felt that high visibility was necessary for good online communication. The personal information visible on Facebook profiles provided more of a sense of trust for these few members, as Rina explained:

“[On WhatsApp] you don’t always know the person’s name. I can see the group’s list of girls here, but I don’t even know who they are. [She pulls out her mobile phone and looks at the list]. Some have names, some have pictures, some have only the phone number. Who are they? Facebook is much more convenient. You can search and find what the person has previously written in groups.”

Some participants had other reasons for preferring Facebook’s high visibility. For some, posting about their condition on Facebook destigmatized PTSD and created a convenient way to vent without feeling that they were a burden to their environment, as Dan expressed:

“As soon as I got past the mental barrier of feeling embarrassed, Facebook enabled me to open up in the most honest and sensitive way. I can choose my words in a way that does not exist in one-on-one interactions. For people who want to open things up and talk, Facebook is very convenient and pleasant. It can get to the level of, sorry to say it in such a way, of having sex with a girl. It’s the most intimate thing there is, and then it’s gone. And I found it to be just what I needed to feel okay. To feel you’re not alone, but also not to become a burden on anyone.”

In addition to having the option to carefully construct a public message without becoming a burden on caregivers, some participants noted that their disclosures facilitated constructive conversations. These conversations led to an attitude change about PTSD in their social networks, as Eldad stated: “You can disclose things to people who are not in the same situation, like friends and family. I think that it’s important for them to hear what’s being said about this topic.” Or, as Barak shared: “I can’t sleep at night nor eat, and I’m in pain. And I’m not ashamed of it. Well, that’s who I am. I shout it out. Whoever feels it’s not suitable—you are welcome to unfriend me”.

In addition to the function of de-stigmatization and venting, Orit emphasized that the high visibility of Facebook served the community as a way to reach out to others like themselves, something that they missed on WhatsApp: “I found out about the WhatsApp group through Facebook. You see, I had no idea the WhatsApp group existed. I got in touch with the WhatsApp group through Facebook Messenger after joining the Facebook group”.

The perspectives presented above highlight how the fear of disclosing personal information, the perceived stigma of PTSD, and the motivation to destigmatize it played significant roles in deciding which platform to use in order to find support. Those who preferred not to expose their own or their partner’s disorder found WhatsApp to be a safer platform to disclose sensitive information. In contrast, those who felt that they were ready to disclose their condition online preferred to take advantage of Facebook’s high visibility. It enabled them to express their thoughts, receive feedback, and reach out to others like themselves in a way that would not burden those close to them. 

### 3.2. Availability

WhatsApp’s high availability level—offering the most efficient way to locate resources when most needed or desired [2]—was a dominant topic in the interviews. Compared to Facebook, WhatsApp offered an immediate source of support. Ben pulled out his phone, touched the screen, and explained: “On Facebook, you first need to get here, and then you have to go in here and look for it [the online support group]. WhatsApp—it’s here. It’s bam, bam, bam. It’s there.” WhatsApp’s high availability also enabled users to receive 24/7 support. Sarah described her search for emotional support from the group over the weekend:

“Last Friday night, I had the feeling of ’this is it.’ Danny was sick, and then he lost it. Being sick is awful to him because then he loses control. And I already felt it coming, and I wrote to them immediately. At 10 o’clock at night, I messaged the group and got instant responses. The group knows what’s going on. Yes, we know each other so well.”

This high availability also enabled members to receive informational support at night. Adam, an admin of a WhatsApp group, shared an example of a question that a group member asked in the middle of the night:

“Someone needed help with a doctor who could prescribe medical marijuana for him. He asked me about it in a direct WhatsApp message, and I posted his question on the WhatsApp group. The responses came in promptly in the middle of the night.”

Nevertheless, some group members found WhatsApp’s high availability to be an annoyance and a detraction. The ongoing connection led some members to experience an information overload, as Barak described: “We need an app that allows you to switch off. If you have a day you can’t handle, you can’t have that ‘ding, ding, ding’ every 15 minutes.” Noya also described why WhatsApp’s high availability had a downside: “I kept reading messages in the group where people explain what needs to be done. I can’t handle this constant advice and replies. I prefer to be in the Facebook group.”

Compared to Facebook, most participants preferred to use WhatsApp because its high availability enabled them to reach out to the group in times of need. The ease of entering the group via the app and the group members’ immediate responses were significant advantages. Nevertheless, a few participants felt that this high availability was burdensome at times and the group’s frequent communications became an additional source of distress. 

### 3.3. Multimediality

The group members highlighted the benefits of providing and receiving information in various configurations such as text, image, audio, and video [46]. Barak described the voice recordings feature as a useful one during tense moments:

“I can’t write during an anxiety attack. I can’t press the keys, they are small and annoying, and I get even more annoyed. I want to break the phone anyway. Instead of destroying your house, you can complain and shout inside the WhatsApp group, and someone will listen to your recording and say, ‘Come on, let’s go to the beach.’ That’s something you can’t do on Facebook.”

Shosh, a member of the partners’ group, found a creative way to use the WhatsApp voice recording feature. She recorded a guided imagery lesson (a stress management technique) and sent it to the group members:

“Last night, someone wrote ‘I need something serious, like some guided imagery.’ I recorded and sent them a script of guided imagery from a class I took. The group’s response was positive: they wrote: ‘Really wonderful, well done really!’ And one member wrote ‘I’m in bed now after listening to your guided imagination class, thank you! Going to fall asleep with a huge smile!’”

Boaz was grieving the loss of one of his comrades (who died in battle) and showed the interviewer a post with a photo he published in the Facebook group about his comrade:

“This is Uri. A unique kid. Blond hair, blue eyes. He was a [unit’s name] fighter. It was tough for me to lose this kid. I grew up with him in Haifa. When he came to the army, I was so happy that he came to [unit’s name]. When he finished his service in [unit’s name], I moved him to my team. I posted his photo and wrote something on the anniversary of his death.”

Group members took advantage of their phones’ multimediality and found new and creative ways to cope with their distress. Nevertheless, some participants were unable to use all of the configurations available because their phone capacity did not allow for such large files. As Ben noted:

“Since I joined this group, my phone has crashed. I have to erase memories all the time, delete things, media, photos, videos.”

The above quotes reveal the ways in which group members used various media configurations to communicate and interact with the groups. 

### 3.4. Surveillance

In their search for support, group admins and group members found WhatsApp to be a useful tool because of its surveillance affordance, defined as the ability to monitor group members’ behavior [47,48]. Surveillance was exercised in two ways: through notifications about message status and notifications about members leaving the group.

***Message status.*** WhatsApp uses a system of ticks to indicate the status of messages that people send. The ticks are visible in the bottom right-hand corner of the message speech bubble, next to the time stamp. One grey tick means that the message was sent successfully, and two grey ticks indicate that the message was delivered successfully to the recipient’s phone. If the two ticks turn blue, then the message has been received/read. Some people disable this function so that others will not track them. However, turning off this function prevents tracking others too. This feature, according to some participants, provided members with a way to check whether their call for help was immediately heard, as Baruch explained: “You will receive an immediate and direct response on WhatsApp. On Facebook, you don’t know if anyone saw it or not.”

Moreover, group admins used the message status affordance to monitor non-active members without bothering them, as Nava articulated:

“Some members are just listeners. No, they don’t have to post messages, only if they want to. It’s not my goal to track these girls, but recently I wanted to see how many members we had in the group. I looked to see who had two blue checkmarks next to my message. Two blue checkmarks means she got the message. That’s great. It means that she’s in the group. She doesn’t have to say anything.”

***Membership status*.** WhatsApp is designed in a manner whereby if a member leaves a group, other group members receive a notification that “[username] left the group.” Nava used this feature to inquire about members’ reasons for departure: “There was a notification that two members had left the group. I called them and asked why they left.” Although the surveillance function was useful, it was not always appreciated by some of the members, as Ben described:

“If I leave the WhatsApp group, I will immediately get a message from the admin, ‘What happened, everything okay?’ If you leave, you have to go through a person. Even if you don’t feel like it, you have to say what’s going on.”

Monitoring group members’ activity was not as common nor, in general, was it technically possible in the Facebook group. When it did take place, it was as a result of members suddenly behaving differently. As Merav described:

“One member occasionally posts in the group. Lately, he publishes posts daily. Something is going on with him. He uploads lots of stuff, photos, personal posts. If I were to tell him that I noticed something different, he would be alarmed by how he exposed himself and say goodbye.”

As the cases above highlight, admins and members can follow members’ activity mainly on WhatsApp by checking their message status. According to participants, this feature offers them a sense of security. They know that in the WhatsApp group, they can check to see who has read their message. The message and membership status notifications offer admins a way to check that a message has reached all participants, even those who don’t respond, and to get in touch with members who have left the group (to make sure they are okay). 

### 3.5. Synchronicity

Study participants pointed to the different levels of synchronous communication—defined as the level of synchronicity in message transmission [49]—that PTSD-OSGs on Facebook and WhatsApp offered them. As with many other forms of text-based online interaction, messages are constructed and sent separately. In practice, participants write different messages at the same time. Compared to the synchronous communication on WhatsApp, some participants found the relatively a-synchronous nature of communication on Facebook beneficial for emotional disclosure, as Noya explained:

“On WhatsApp, you get lost. You send a message, someone responds to you, but someone else sends a new message and pushes yours back. It’s a little hard to follow. On Facebook, you say something, and people relate to you. You can later respond to others’ comments, but everything is in the same thread. There is something more embracing, more personal about it.”

Thus, participants found that WhatsApp offered fewer options for organized correspondence because of its synchronous affordance. Nava, a member of the partners’ group, pulled out her phone and showed the interviewer what she did when her response was pushed back: “I went to the message that I wanted to return to, clicked on it, and replied. Today, everyone knows how to use this function. That message was sent at 10 a.m., and I responded to it at 6 p.m. That’s great. It’s an ongoing chat.”

When considering the different synchronicity levels afforded by WhatsApp and Facebook, WhatsApp synchronous communication seemed to serve as a hindrance; that is, participants felt that their call for emotional support was left unanswered because of the too-prompt interactions in the group. Participants preferred the asynchronous and turn-taking type of interaction Facebook offered them for more organized forms of communication.

## 4. Discussion

This study applied an affordance framework to examine how digital platforms can assist military veterans (who cope with PTSD) and their partners searching for online support and how these platforms can hamper their coping behaviors. The findings have practical implications for both healthcare professional and researchers. 

For healthcare professionals, the study offers insights about what it means to cope with PTSD in the digital age. Communication technologies are ubiquitous in contemporary life and, as demonstrated throughout the findings, their use extends to coping with PTSD. Understanding how and why these technologies are used by people who cope with PTSD can be of great value to healthcare professionals. The affordances underscore those qualities of communication technologies that are specifically significant for people with PTSD and may be leveraged to enable coping efforts. In better understanding how communication technologies are perceived and used, healthcare professionals can support and guide people with PTSD in engaging in digital coping efforts. Understanding the various types of affordance that exist will enable healthcare professionals to identify their potential benefits and pitfalls and advise patients accordingly.

For researchers, this study advances our understanding of how technologies are used among people who experience a potentially stigmatizing condition. Whereas digital coping models [4] offer a broad perspective for explaining digital coping, this study elucidates the connection between a specific stigmatizing mental health disorder (i.e., PTSD) and perceptions of communication technologies (i.e., affordances), and specifies the uses of technologies for coping with this mental health disorder. As such, this study promotes our understanding of health stigma and stigma management communication. 

### Limitations

The affordances identified here represent early efforts to define and apply what is afforded by WhatsApp and Facebook to PTSD support groups. Appropriate targeting of a given platform to a participant group—in this case, people coping with PTSD—is critical. However, as in any case-study research design, the data collected cannot be generalized to the broader population. Moreover, the conceptualization of affordances is a continuing, iterative process across many research disciplines. In the affordance framework, the definition and the scope of each affordance has been contested [47]. Nevertheless, this ongoing debate is evidence of the value of this framework. Even if not perfect, it allows for a mindful selection through a far-reaching and ever-evolving technological environment.

## 5. Conclusions

As indicated in the study’s findings, understanding the relation between PTSD characteristics and how communication affordances can fit specific support needs advances our theoretical understanding of the interplay between people and technology. Moreover, this study represents an opportunity in the area of technology. Digital intervention designers need to be mindful of the affordances of the products they develop. Target groups’ thoughtful articulation of needs, and an assessment of platform affordances that address these needs, may yield a complementary partnership that can lead to better health outcomes.

## Figures and Tables

**Table 1:** Participants’ information.

Name	Gender	Role	Age	Education Level	Marital Status	WA	FB
Adam	Male	PTSD	70	B.A.	Divorced	*	*
Avi	Male	PTSD	52	B.A.	Married	*	
Merav	Female	PTSD	63	Post-secondary	Divorced		*
Amos	Male	PTSD	41	Secondary	Married	*	
Asher	Male	PTSD	55	B.A.	Married	*	
Aryeh	Male	PTSD	77	B.A.	Married		*
Barak	Male	PTSD	37	Secondary	Married	*	*
Azriel	Male	PTSD	34	Secondary	Married	*	*
Baruch	Male	PTSD	35	Post-secondary	Married	*	*
Ben	Male	PTSD	37	Post-secondary	Married	*	*
Boaz	Male	PTSD	48	B.A.	Married		*
Mia	Female	PTSD	30	Secondary	Single	*	
Chaim	Male	PTSD	39	B.A.	Married	*	*
Dan	Male	PTSD	29	B.A.	Single		*
David	Male	PTSD	34	B.A.	Married	*	
Daniel	Male	PTSD	41	Post-secondary	Married		*
Doron	Male	PTSD	64	B.A.	Married		*
Ehud	Male	PTSD	68	B.A.	Divorced		*
Eldad	Male	PTSD	65	Primary	Married		*
Eliezer	Male	PTSD	40	Secondary	Married	*	*
Miriam	Female	PTSD	32	M.A. or above	Married	*	*
Ezra	Male	PTSD	63	Secondary	Married		*
Gad	Male	PTSD	65	Secondary	Married	*	*
Natalie	Female	PTSD	32	M.A. or above	Single	*	*
Gershon	Male	Facilitator	55	B.A.	Married	*	
Nurit	Female	Partner	30	M.A. or above	Married	*	*
Nava	Female	Partner	58	M.A. or above	Married	*	
Noya	Female	Partner	38	Secondary	Married	*	*
Orit	Female	Partner	38	Post-secondary	Married	*	*
Rachel	Female	Partner	48	M.A. or above	Married	*	*
Rina	Female	Partner	29	M.A. or above	Married	*	
Sarah	Female	Partner	53	B.A.	Married	*	*
Shira	Female	Partner	60	Secondary	Married	*	
Shosh	Female	Partner	48	B.A.	Married	*	

Note: WA = WhatsApp group member. FB = Facebook group member. * = Digital group member.

**Table 2:** Thematic categories.

Affordances	Themes	Participants’ Quotes
Visibility	Public nature of posts	I know some say WhatsApp is private. That’s bullshit. You have to be very careful and even more careful what you write on Facebook. I am very careful not to write anything suicidal.
	Disclosing PTSD condition	Lots of people commented on my first post. It filled me with lots of energy. On the other hand, it stressed me terribly cause I’m not used to this level of exposure.
	Anonymity	There is no anonymity. Not in the WhatsApp group and not on Facebook. I tried to make our Facebook group as anonymous as possible. If you’re not a member, but you’re looking for the group, you can’t see the posts published, but you can see who the members are.
Availability	Immediacy	I can upload a post at 3 am and know that there will be immediate comments. Lots of group members are awake, and they’ll respond immediately.
	Reach	One member needed help with a doctor who would write him a prescription for medical marijuana. So I asked the group in the middle of the night and lots of people shared their experience.
Multimediality	Use of voice messages	If something happens at home, I record a message, share it with the girls, and then their reactions are encouraging.
	Use of video	In one video some veterans filmed themselves telling their story to raise awareness and raise donations for the organization.
	Use of images	Sometimes I post photos of what needs to be done at night to go to sleep. Combinations of alcohol and drugs. Any possible way to sleep more than two hours without getting up and screaming.
Surveillance	Admins monitoring members	I was upset and left the group. Then the admin and another member wrote to me and convinced me to come back.
	Members monitor members	If someone leaves the group, suddenly there is complete hysteria. And the biggest panic is when someone writes, “Enough is enough” because he can kill himself within 15 min.
(A)synchronicity	Ongoing communication	Once people felt comfortable in the offline group, they continued connecting on the WhatsApp group and kept talking about the training.
	Discontinuous communication	Communication on Facebook works like movie cuts. A few days after writing about my trauma, I can upload entirely different content, like a selfie with my nephew, and no one remembers I wrote something hard and painful.
	Controlling communication	On Facebook, we have control. We can look away when we feel like it, and we do not have to give in to any norms. Facebook is not invasive or intrusive. It’s nothing.

## Data Availability

Not applicable.
